# Sacro-Iliac Diseases

**Published:** 1908-08

**Authors:** C. R. Andrews, Michael Hoke

**Affiliations:** Atlanta, Ga.; Atlanta, Ga.


### Journal-Record of Medicine

Successor to Atlanta Medical and Surgical Journal, Established 1855, and Southern Medical Record, Established 1870.

OWNED BY THE ATLANTA MEDICAL JOURNAL CO.

Published Monthly.

*Official Organ Fulton County Medical Society, State Examining Board, Presbyterian Hospital, Etc.*

EDGAR G. BALLENGER, M. D., Editor.
BERNARD WOLFF, M. D., Supervising Editor.
A. W. STIRLING, M. D., C. M., D. P. H., J. S. HURT, B. Ph., M. D., Associate Editors
E. W. ALLEN, Business Manager.

COLLABORATORS

DR. W. F. WESTMORLAND, General Surgery.
F. W. McRAE, M. D., Abdominal Surgery.
H. F. HARRIS, M. D., Pathology and Bacteriology.
E. B. BLOCK, M. D., Diseases of the Nervous System.
MICHEL HOKE, M. D., Orthopedic Surgery.
CYRUS W. STRICKLER, M. D., Legal Medicine and Medical Legislation.
E. C. DAVIS, A. B., M. D., Obstetrics.
E. G. JONES, A. B., M. D., Gynecology.
R. T. DORSEY, Jr., B. S. M. D., Medicine.
L. M. GAINES, A. B., M. D., Internal Medicine.
J. N. LeCONTE, M. D., Diseases of the Stomach and Intestiness.
L. B. CLARKE, M. D., Pediatrics.
EDGAR PAULIN, M. D., Opsonic Medicine.
R. R. DALY, M. D., Medical Society.
A. W. STIRLING, M. D., Etc., Diseases of the Eye, Ear, Nose and Throat.
BERNARD WOLFF, M. D., Diseases of the Skin.
E. G. BALLENGER, M. D., Diseases of the Genito Urinary Organs.

VOL. X. AUGUST, 1908. No. 5

### SACRO-ILIAC DISEASES.*

BY C. R. ANDREWS, M. D., AND MICHAEL HOKE, M. D.,
ATLANTA, GA.

In considering the sacro-iliac synchondroses, it must be remembered that we are dealing with true joints which are capable of distinct motion, but which, unlike the other joints of the skeletal structure, consist of flat, bony surfaces which are opposed in oblique axes. In consequence of this fact, the stability of these articulations is dependent upon the strength and tone of the muscles and ligaments which act doubly by preserving an anatomic relation and protecting them.

The pelvic girdle may be considered as the structural base for the skeleton, having attached to it the strong trunk muscles as

*Read before the Georgia Medical Association, Fitzgerald, April 15 16-17, 1908.

well as all the important muscles of the thigh. Hence it is evident that any instability of the pelvic articulations must interfere with the proper action of the muscles thereunto attached, and conversely it is true that any lack of development or alteration in tone of the muscles and ligaments, which protect and hold in position the pelvic joints, must likewise render impossible their normal anatomic relation, and with change of anatomic relation we have impaired function.

ANATOMY.

The articular surfaces of the sacro-iliac synchondroses are broad and flat, the obliquity of their axes being in two directions, upward and outward and forward and outward. A consideratlon of the differences between the narrow approximation of bony surfaces forming the pelvic articulation, and the broad, extensive opposition between the sacrum and the ilium will clearly demonstrate that the stability of the pelvic girdle is dependent upon the sacro-iliac articulations and not the simphysis pubis. As a matter of fact, in cases of extrophy of the bladder, the pubic bones may be absent without seriously interfering with locomotion.

Motion is a normal function of the sacro-iliac joints and consists of a tilting of the sacrum on the ilia or the ilia on the sacrum. The axis of this motion is a transverse one, situated about the middle of the sacrum. It is obvious that in tilting, when the promontory of the sacrum moves forward, the tip moves backward, and vice versa.

Certain physiological conditions bear a direct relation to relaxation of the sacro-iliac joints. It is a well known fact that there is a demonstrable amount of relaxation during pregnancy, which becomes more marked toward delivery. This relaxation may not be sufficiently marked to deserve attention, while on the other hand it may be so great as to cause serious inconvenience as regards pain and discomfort, and materially interfere with locomotion, requiring treatment both during pregnancy and after delivery.

During menstruation, which physiologically may be considered as a mimature pregnancy, there is often a relaxation of the joints in question. This unquestionably explains many of the backaches occuring at the menstrual period, which may often require mechanical support for their alleviation.

During pregnancy and menstruation, congestion of the pelvic viscera is present, and since these conditions may cause relaxation of the sacro-iliac points, Goldthwait suggests that abnormal congestion of the pelvic organs might cause relaxation, and conversely long standing relaxation of the sacro-iliac synchondroses might induce congestion of the pelvic viscera. It has been shown that these joint conditions often improve after plastic operations on the perineum and cervix, and removal of uterine tumors.

From an obstetric viewpoint the movements of the sacrum are of importance. If the upper part of the sacrum be tilted backwards, the anterior-posterior diameter of the brim of the pelvis is increased, and on account of the double obliquity of the articular surfaces, the lateral diameter is likewise increased. But, when in tilting, the axis of motion, passing through the center of the sacrum, as the diameters of the pelvic brim are increased, the tip of the sacrum moves forward and the diameters of the outlet are decreased correspondingly. A knowledge of these facts is often of value to the accoucheur, since the pressure necessary to produce these sacral movements is not at all unbearable by the patient.

Again, since the trunk muscles, which are attached to the pelvis, play such an important part in delivery, the auxillary expulsive force contributed by the trunk muscles is greatly interferred with if the sacro-iliac joints be relaxed, because in that case the basal attachment of these muscles is insecure. This insecurity may be relieved at the time of delivery by a tight fitting belt or bandage about the trochanters.

In the consideration of this subject it must be remembered that we are dealing with true joints, which are subject to the same diseases as joints in other parts of the body: Tuberculosis, maligancy, injury, toxic inflammations, etc. But it is the desire of the authors to draw particular attention in this imperfect exposition of the subject, to the condition of relaxation and chronic strain, often due to faulty posture associated with toxic inflammation. When we remember that these joints consist of flat, bony surfaces with articular axes in an oblique direction, with no bony projections, and held together only by muscles and ligaments, it is not surprising that chronic strain and relaxation are so common. Ligamentous and muscular support are quite less ineffectual than where the heads of bones are fitted into globular cavities, or the articular surfaces superimposed upon each other.

It has been observed by us many times that with an existing basal condition of excessive intestinal putrefaction, any injury or undue strain to a joint may precipitate a toxic inflammation. And since the sacro-iliac joints are liable to injury and strain, toxic inflammation of these joints is rather common. This superimposed toxic process is of great importance, since, for the relief of the sufferer, mechanical measures must not only be adopted, but also consideration must be given to the intestinal condition.

The mildest manifestation of disturbance in these joints is a slight strain or discomfort in faulty attitudes, as for example, in stooping. The muscles protect the joints for a while, but as these tire the strain is put upon the ligaments, and as these begin to relax and become tense, discomfort is felt. This is usually relieved by stretching as in so doing the lumbar spine is drawn forward, carrying forward also the sacrum into its normal position, the strain thereby being relieved. Downward strain of these joints is also possible from long standing and is relieved by a change of attitude which brings other muscles into play. Strain may also occur in the recumbent position, the spinal muscles finally tiring and the lumbar spine sagging, carrying backward with it the upper part of the sacrum. After anaesthetics where relaxation has been complete and the patient lying on a hard table which cannot adapt itself to the shape of the back, the lumbar spine sags greatly and it is in this way that the great majority of postoperative backaches are explained. Such backaches are easily controlled by supporting the lumbar spine with a pillow, and holding firmly together the sacro-iliac articulatlons with a belt, bandage or adhesive strapping. Backaches occur in the same manner in acute illnesses where the general muscular system becomes relaxed and the individaul, lying largely on the back, the lumbar spine is permitted to sag.

The same condition of affairs occurs from long sitting if the body is not held erect but is permitted to "slouch," the lumbar spine becomes flexed, carrying the base of the sacrum backward and straining the sacro-iliac ligaments.

In all these conditions previously described, excepting the toxic inflammations, there has been no actual damage done the joints. The condition of strain and accompanying symptoms have been relieved with change of position. If, however, strain is continued until the bones are displaced, or if the strain be sudden and sufficiently severe to lacerate some of the ligaments, we have a true sprain, accompanied by a sharp pain or "stitch" in the back, with the pain oftimes referred to one or the other of the sacro-iliac joints. Lumbago is very often simply a manifestation of a sprain of a sacro-iliac joint.

When a sprain with displacement occurs, the displacement is often reduced when the correct attitude is assumed. But if the small irregularities on the articular surfaces of the sacrum and the ilia catch, then manipulation may be necessary for reduction.

Again, if the deforming force be moderate in severity and continued over a long period of time, a condition of "chronic relaxation" is reached, with or without displacement. Great discomfort and disability occur in this condition incident to the instability of the pelvis, due to relaxation of the sacro-iliac ligaments. The attachment of the thigh and trunk muscles to the pelvis, explains any disability that may occur in such a condition.

The sacral plexus of nerves containing branches from the lumbar plexus is situated just in front of the sacro-iliac articulations, and it is obvious that strain or displacement of these joints may produce irritation in the nerve trunks situated anteriorally to them. The symptoms resulting in this way are dependent on the nerve trunks injured and the severity of the injury. In this way, also, referred pains in the lower extremities an dareas of anaesthesia and hyperesthesia are explained. Of greater importance, however, are obstinate sciaticas which, in the vast majority of cases, are due to irritation produced by the sacro-iliac synchondroses. Dr. Goldthwait tells me that nine cases out of every ten of sciatica are produced in this way, and its importance as regards treatment is quite evident. He states that many cases of sciatica cannot be cured unless the sacro-iliac joints are properly treated. Since it is a well established fact that when a nerve is injury, the futility of treatment applied to the seat of pain is quite evident.

When displacement occurs, it is most often backward with reference to the upper part of the sacrum. In acute conditions this displacement is usually unilateral, while in the cases of long standing "chronic relaxation," both articulations are involved resulting in the flat back so commonly seen.

Forward displacement of the sacrum, however, is possible, and in falling from great heights and striking on the feet, it may actually be driven downward with the ilia raised and the legs apparently shortened.
illiac joints, limitation of motion may be shown by motions of the body on the thighs, or by motions of the thighs on the body. If the knees be held stiff and the body bent forward, the amount of forward bending will be limited if the sacro-iliac joints be at fault. This motion is made by the hips and spine up to the point where the hamstring muscles become tense,then it is made by the sacro-iliac joints, hips and spine. When the hamstring muscles become tight and strain is put on the irritable sacro-iliac articulations, the spinal muscles are thrown into reflex contraction and the motion is limited.

The way in which to determine whether this forward limitation of motion is due to the synchondroses or to the spine, is to relieve the tension on the hamstring muscles by permitting the patient to sit, when forward bending will be much freer.

When forward bending is limited, lateral bending is likewise affected, and since one side is usually more affected than the other, a difference wil lexist between the amount of bending toward the two sides. In standing there will often be a marked lateral deviation of the body away from the affected side. Since the hamstring muscles play no part in lateral bending, no difference is noted whether the patient assume the sitting or standing posture.

Backward bending is usually very guarded, and cases where the body is thrown forward and held in that position, backward bending is, of course, impossible.

Nerve irritation with referred pain is most common in cases of sudden unilateral displacement. The pain may vary from a slight twinge to one severe in character. When the relaxation is slow and the displacement is gradual, the nerve trunks adapt themselves to these changes and referred pain is not so common.

Of the infectious processes occuring in these joints, the nontubercular are more common than the tubercular, though both are very rare. Toxic inflammations, however, are quite common, the resultant damage varying from a few adhesions to complete ankylosis. Toxic inflammation may occur as a part of a general polyarticular involvement.

Proper attitude is dependent on normal muscular tone and the security of the base to which the trunk muscles are attached. If this be true, it is evident that with an insecure pelvis, correct posture cannot be assumed without undue muscular effort. With a pelvic girdle whose joints are relaxed, the body droops forward, in which condition the support given the abdominal viscera by the trunk muscles is removed and the abdominal organs sag. In the treatment of these various ptoses due consideration must be accorded the pelvic girdle if the ligaments which hold it secure are relaxed or if, for any reason, it be insecure.

*Symptoms*—The most important symptom to be considered in the diagnosis of this condition is limitation of motion. If the sacro-illiac joints be deceased or strained, any motion which causes strain will be limited involuntarily, as is exactly the case with other joints of the body when diseased. In the case of the sacro-

These tests can also be carried out under conditions in which the sacro-illiac points do not carry the body weight. To accomplish this, the patient lies on his back. Instead of forward bending, the legs are raised, the knee being half stiff. For lateral bending the legs are abducted and for backward bending, the patient lies on the face and the legs are hyperextended. With the patient lying on the back and with the knee bent, all normal motions will be unlimited and painless unless the joint be very sensitive or unless extremes of motion be carried out; outward rotation and abduction are then usually painful.

If only one joint be involved, straight leg raising will be limited on both sides though naturally not so much so on the unaffected side. Pain will also be referred to the affected side no matter which side is raised. The explanation of this is that when the leg on the unaffected side is raised, the hamstring muscles become tense and move the ilium on that side, naturally the sacrum is carried with it and consequently we have motion also on the affected side.

*Pain*—With the exception of limitation of motion, pain is the most important diagnostic symptom in this malady. Pain may be referred directly to the ancro-iliac joints, but more often to the sacral region, also to the leg and foot. The pain which is referred to the leg and foot may be localized in definite areas. In these cases, pressure over the nerve course elicits no pain, a diffrential diagnostic point from neuritis.

The pain present is usually worse at night, dependent upon the increased strain which results from recumbency, and is also made worse by any motion or position which increases strain.

In the case of children, the pain most usually takes the form of legache or backache. While it may be present during the day, it is almost always present at night, and is frequently mistaken for so-called growing pains, and sometimes confused with the night cries of tuberculous hip disease.

*Swelling*—There is rarely ever swelling in sacro-iliac lesions unless we have to deal with toxic inflammations, tuberculous and non-tuberculous infections.

*Abnormal Motility* of the synchondroses is present in many cases of sacro-illiac disease due to long continued strain. This increased motion is discoverable by several tests, namely, by forced hypehextension of the thighs one at a time, moving the ilium on the sacrum; with the patient standing, hold one hand over the sacrum and grasp the pubic bones with the thumb and forefinger of the other hand, the legs are then raised one at a time. Again, by grasping the iliac crests with the fingers and holding the thumbs over the sacro-iliac joints, and having each leg raised separately. Still another, by having the patient lie on the back, placing one hand over the synchondroses, the patient raising the legs one at a time.

While there may be actual abnormal motility in the synchondroses proper, motions which increase the strain upon the ligaments of these joints may be strictly limited.

*Attitudes*—On standing the lumbar curve will be ibliterated if the sacrum is diplaced backward, the reverse being true if sacral displacement is forward. The whole body is often thrown forward. The body is usually inclined away from the diseased joint. When rising from the recumbent to the upright position, the spine is held rigid and the hands often used for support. Flexion of the trunk is avoided. Walking is guarded if the condition be acute, and a long step is imposisble, due to spasm of the hamstring muscles. If such instability is present, a rolling or waddling gait is seen. In lifting weights or stair climbing, the hands are often used for support.

*Treatment*—When in the synchondroses either acute or chronic strain exists, naturally the first indication is to relieve this strain and correct conditions which are instrumental in its production. Long satnding should be avoided, and in standing the correct attitude should always be maintained. When sitting, lounging should not be indulged in, and when recumbent, a firm pillow should be placed under the hollow of the back to prevent the lumbar spine from sagging.

In many cases simply relieving the attitude of strain will relieve the strain and nothing else will be needed. But in others, while this relieves, it does not cure, and mechanical apparatus must be devised for the support of the joints. Adhesive plaster straps across the lower back and buttocks, or belts so applied as to support the sacro-iliac joints, combined with a low corset to corerct lumbar flexion, will suffice in some cases, but in others plaster-of-paris casts are necessary and more complicated forms of apparatus will have to be devised.

In some cases where the symptoms are due to joint strain, mechanical support will only be palliative. Muscular and ligamentatious weakness are the fundamental aetologic factors, for the correction of which, massage, stimulating baths, and special exercises are required.

When sacral displacement is present, the upper part of the bone is most usually pushed backward and rarely apparently downwards. In this condition the bone can almost always be replaced by hyperextending the spine, or by placing the patient upon the back and raising the leg with the knee straight.

A procedure recommended by Goldthwait is "to have the patient lie face downward with the thighs and legs supported upon one table and the head and shoulders upon another, the body hanging entirely unsupported between. The plaster jacket may be applied in this position."

In other cases of subluxation in order to produce complete muscular relaxation, an anaesthetic is necessary and oftimes more definite pressure is required directly on the sacrum.

In the forward subluxations, flexion of the spine and hyper extension of the thighs is the correct manipulatory procedure. In some cases it will be necessary to manipulate the ilium on the sacrum. In cases where extreme relaxation is present, recumbency in a plaster jacket will be required.

Recent injury requires the same treatment as strain except that recumbency will often be indicated. Later the patient may be allowed up as the symptoms of judgment dictate. In this type of cases some form of apparatus will have to be worn for several months, followed by massage, exercise, baths, electricity, etc. In cases of strain and subluxation, some form of apparatus should be worn for about three months, followed by the above mentioned methods of strengthening muscular and ligamentous support.

In women a broad belt of webbing attached to the base of the corset and strengthened by steels is very efficient. Woven elastic trunks, fitted about the thighs and buttocks, often prove satisfactory. In stout women the Osgood brace is very efficacious.

A firm pillow under the lumbar spine or some form of apparatus will usually have to be worn at night, as during recumbency the pain is often great.

The fact to which the writers particularly desire to call attention is that toxic inflammation due to excessive intestinal putrefaction may be superimposes upon any of the condition of the sacro-iliac joints herein described, and in order for a cure to be effected, appropriate treatment must be accorded the disturbed digestive function as well as the diseased joints.

The prognosis is good if treatment be extended over a sufficiently long period of time.

